# Detection of Pharmaceutical Contamination in Amphipods of Lake Baikal by the HPLC-MS Method

**DOI:** 10.3390/antibiotics13080738

**Published:** 2024-08-06

**Authors:** Tamara Y. Telnova, Maria M. Morgunova, Sophie S. Shashkina, Anfisa A. Vlasova, Maria E. Dmitrieva, Victoria N. Shelkovnikova, Ekaterina V. Malygina, Natalia A. Imidoeva, Alexander Y. Belyshenko, Alexander S. Konovalov, Evgenia A. Misharina, Denis V. Axenov-Gribanov

**Affiliations:** Research Department, Biological and Soil Faculty, Irkutsk State University, Irkutsk 664003, Russia; telnovatamara1410@gmail.com (T.Y.T.); marymikhmorg@gmail.com (M.M.M.); chiffa.tap@gmail.com (S.S.S.); vlasovafippo23@gmail.com (A.A.V.); marriee.dmitrieva@gmail.com (M.E.D.); shelkovnikova551@gmail.com (V.N.S.); cat.malygina@gmail.com (E.V.M.); nat.imidoeva@gmail.com (N.A.I.); al.belyshenko@gmail.com (A.Y.B.); alexander.s.konovalov.87@gmail.com (A.S.K.); me603@mail.ru (E.A.M.)

**Keywords:** amphipods, antibiotics, Baikal, HPLC-MS, ibuprofen, pharmaceutical pollution, endemics

## Abstract

Pollution by active ingredients is one of the most significant and widespread forms of pollution on Earth. Medicines can have a negative impact on ecosystems, and contamination can have unpredictable consequences. An urgent and unexplored task is to study the Lake Baikal ecosystem and its organisms for the presence of trace concentrations of active pharmaceutical ingredients. Our study aimed to conduct a qualitative analysis of active pharmaceutical ingredients, and quantitative analysis of ibuprofen in endemic amphipods of Lake Baikal, using methods of high-performance liquid chromatography and mass spectrometry (HPLC-MS). Acetylsalicylic acid (aspirin), ibuprofen, acetaminophen, azithromycin, dimetridazole, metronidazole, amikacin, spiramycin, and some tetracycline antibiotics were detected in the studied littoral amphipods. We also detected different annual loads of active pharmaceutical ingredients on amphipods. Using the multiple reaction monitoring (MRM) mode mentioned in GOST International Technical Standards, we detected molecules, fragmented as amikacin, chlortetracycline, doxycycline, oxytetracycline, dimetridazole, metronidazole and spiramycin. Thus, we first revealed that invertebrates of Lake Baikal can uptake pharmaceutical contaminants in the environment.

## 1. Introduction

Pollution of water bodies by various toxicants, pesticides, or drugs is one of the most significant environmental problems [1]. It is known that drugs can have negative effects on ecosystems, and pollution can cause unpredictable consequences in both the short and long term [2].

Pollution from active ingredients is closely linked to the increasing consumption of drugs by people [3]. This is fueled by such factors as demographic aging, high prevalence of chronic diseases, and the availability and emergence of new drugs. The nonregulated consumption of drugs by mankind leads to the penetration of drugs into domestic wastewater in unchanged form or as active metabolites [4].

Antimicrobial, antitumor, and antiviral drugs are top sellers in the global pharmaceutical market. Antibiotics are recognized as a group of drugs acting as environmental pollutants [5]. Previous studies have shown the presence of antibiotics in rivers [6,7], lakes [8,9], groundwater [10], invertebrates [11], and other environments. The mass and uncontrolled use of antibiotics contributes to a decrease in their effectiveness and the development of antibiotic resistance [12,13], increasing the burden on natural ecosystems.

Several studies have shown that drugs such as neomycin, trimethoprim, sulfamethoxazole and enrofloxacin have a high degree of toxicity against test cultures of *Vibrio fischeri*, *Daphnia magna*, *Moina macrocopa* and *Oryzias latipes.* Sulfamethazine, oxytetracycline, chlortetracycline, sulfadimethoxine and sulfathiazole are moderately toxic to organisms. Ampicillin and amoxicillin are the least toxic to the above organisms [14].

Drugs related to the non-steroidal anti-inflammatory class are also known to be toxic to inhabitants of aquatic ecosystems [15,16]. Diclofenac, ibuprofen, acetylsalicylic acid, naproxen, and acetaminophen have been reported to be present in different water bodies [17,18] as pharmaceutical pollutants. For example, acetaminophen has been detected in wastewater in Europe [19], USA [20], and the River Tyne (UK) [21]. Ibuprofen has been detected in the Llobregat River (Spain) [22], the Pearl River Delta (South China) [23] and in other locations by other publications.

Given this background information, effects of pharmaceutical pollution on ancient aquatic ecosystems may be dangerous, but are little studied. As hypothesized, active ingredients of drugs can reduce the biological diversity of endemic organisms that are highly sensitive to pharmaceutical agents [24]. Lake Baikal is an ancient water body and is a lake of tectonic origin in southeastern Siberia. The lake age is typically cited in the literature as 25–30 million years. However, extensive geological studies suggest that the geological age of Baikal is not less than 60 million years [25]. Baikal is the deepest lake on the planet and the largest natural reservoir of freshwater [26,27,28]. Currently, Lake Baikal is home to more than 2600 animal species and has a high rate of endemism [29]. The Baikal ecosystem is characterized by its structural and ecological complexity.

An urgent and unexplored task is to study the Lake Baikal ecosystem and its organisms for the presence of trace concentrations of active pharmaceutical ingredients. Notably, no monitoring observations regarding the detection of drugs in Baikal zoobenthos have been conducted to date. Invertebrate animals inhabiting the littoral zone of the lake are the first to encounter different pollutants and can accumulate them, thereby facilitating the migration of active ingredients along the food chains.

The aim of this study was to assess the contamination of Lake Baikal amphipods with active pharmaceutical ingredients using high-performance liquid chromatography and mass spectrometry.

## 2. Results

During the first stage of the study, we estimated the retention times of standard analyte solutions and achieved simultaneous detection of five analyzed components by selecting chromatographic conditions. The peak retention time for aspirin was 4.6 min, for azithromycin—2.9 min, for ibuprofen—5.7 min, for acetaminophen—0.5 min and for tetracycline—2.6 min. The variance of retention time was 0.05–0.07 min. Table 1 shows data on the presence of trace concentrations of active pharmaceutical ingredients in individual amphipods of *Eulimnogammarus verrucosus* species collected near Bolshoe Goloustnoe settlement in August 2020. Aspirin was detected in 10 out of 16 analyzed samples (level of contamination 63%; mean 0.62; SD 0.12; variance 0.25). Azithromycin and ibuprofen were found in 9 and 12 samples, respectively.

**Figure 1:**
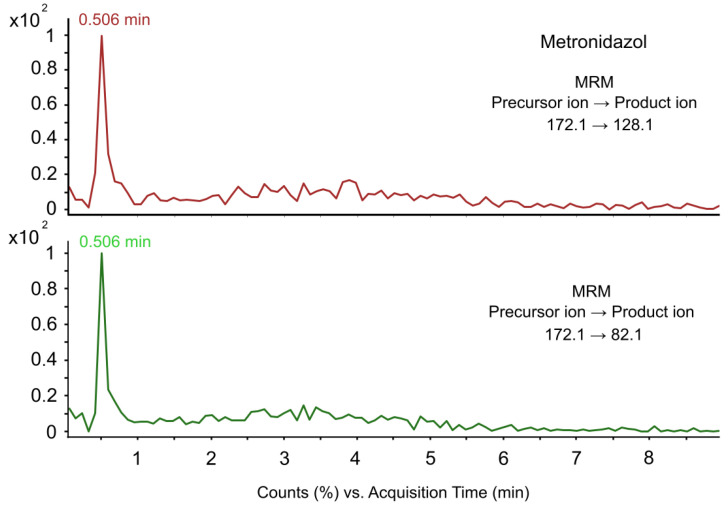
Chromatogram of metronidazole detected in samples of amphipod *E. verrucosus* (2020).

Then, we performed additional screening of drugs whose content in food products is regulated by GOST. The results of these analyses are presented in Table 2. The following precursor and product ions of drugs were detected in all 16 samples of amphipod species *E. verrucosus*: chlortetracycline, doxycycline, oxytetracycline and metronidazole. Selective molecular ions of amikacin, dimetridazole and spiramycin were detected in several samples of amphipods. Molecular ions of dimetridazole were detected in 11 out of 16 samples (level of contamination: 69%; mean 0.69; SD 0.12; variance 0.23). Molecular ions of spiramycin were detected in 4 samples (level of contamination: 25%; mean 0.25; SD 0.1; variance 0.20). Ions of amikacin were detected in 15 out of 16 samples (level of contamination: 94%; mean 0.93; SD 0.06; variance 0.06). Figure 1 shows the chromatograms of metronidazole, including the chromatograms of precursor and product ions. The typical raw data chromatograms of these analyses are presented in Supplementary Materials S1, Figures S1–S9.

At the same time, samples of *E. verrucosus* did not reveal selective molecular ions of the following substances: erythromycin, clarithromycin, tylosin, lincomycin, ampicillin, amoxicillin, florfenicol, thiamphenicol, sulfamethazine, sulfapyridine, gentamicin, hygromycin B, apramycin, and demeclocycline (Supplementary Materials S1, Figure S10).

**Figure 2:**
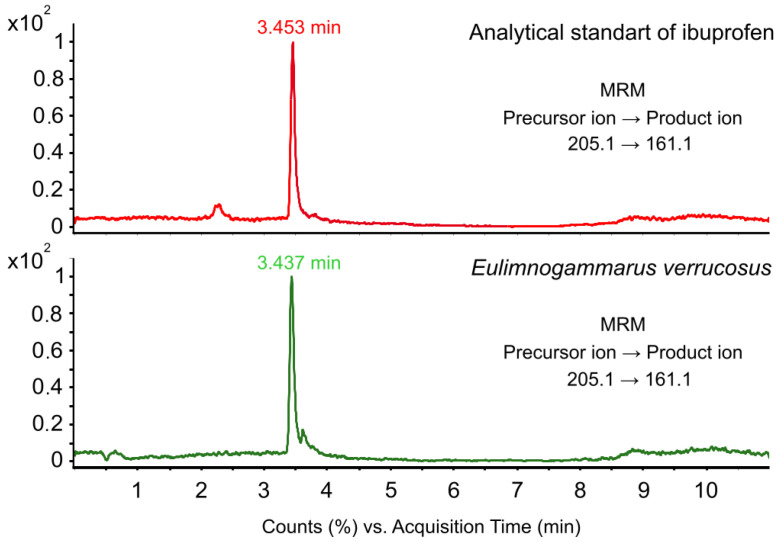
Chromatogram of ibuprofen detected in samples of amphipod *E. verrucosus*.

The monitoring conducted at the same sampling point in 2020 and 2022 revealed differences in the pharmaceutical contamination of amphipod species. Based on the analysis of ibuprofen (Figure 2), we detected that almost 70% of *E. verrucosus* were contaminated in 2020, and 27% of *E. verrucosus* specimens were contaminated in 2022 (Tukey’s test *p* < 0.002; *t*-test *p* < 0.003; Kruskal–Wallis *p* < 0.003). Analysis of other pharmaceutical pollutants using MRM or selective mode was not performed in 2022.

**Figure 3:**
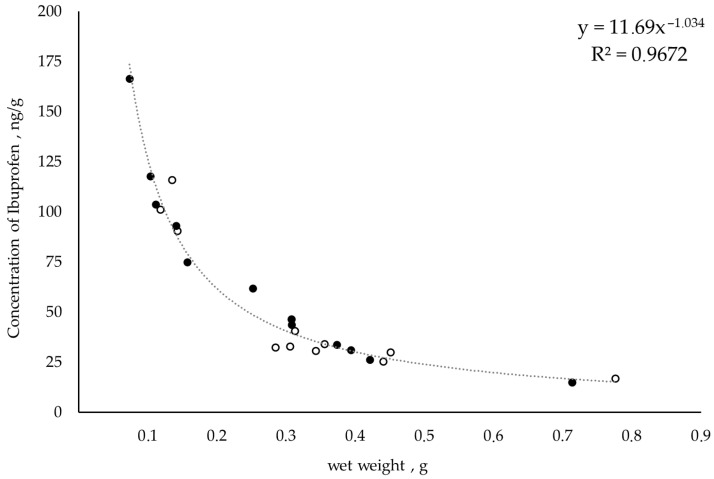
Correlation between concentration of ibuprofen and the wet weight of species *E. verrucosus* amphipods, ng/g (white marker—2020; black marker—2022).

The analysis revealed a significant relationship between the concentration of ibuprofen and the wet weight of *E. verrucosus* amphipods. The concentration of ibuprofen was found to be higher (*p* < 0.0001–*p* < 0.0004, two-way ANOVA) in small (juvenile, 0.1–0.2 g) amphipods than in the big (adult, 0.5–0.8 g) animals (Figure 3). Thus, *E. verrucosus* amphipods with up to 0.2 g wet weight contained ibuprofen in concentrations ranging from 74.71 to 166.38 ng/g. Amphipods with a wet weight of 0.2 to 0.5 g contained ibuprofen in concentrations ranging from 18.50 to 61.75 ng/g. Finally, amphipods with a wet weight above 0.7 g were characterized by ibuprofen concentrations in the range of 14.92–16.98 ng/g.

## 3. Discussion

This study confirmed the hypothesis regarding contamination of Lake Baikal zoobenthos representatives, using amphipods as an example, with active pharmaceutical ingredients, or their selective molecular ions. Contamination of the Lake Baikal ecosystem and its amphipods by active pharmaceutical ingredients is a significant and urgent problem. However, only a small number of studies have been published to date that characterize pollution of Lake Baikal by active pharmaceutical ingredients.

Studies conducted by Meyer M. F. et al. (2021, 2022) found the presence of pharmaceutical agents such as acetaminophen, paraxanthine, caffeine, cotinine, cimetidine, diphenhydramine, phenazone, and sulfachloropyridazine in Lake Baikal water. Other ingredients, such as amphetamine, morphine, carbamazepine, tenamphetamine, methamphetamine, thiabendazole, methylenedioxymethamphetamine, sulfamethazine, sulfamethoxazole, and trimethoprim were not detected [30,31].

Our study shows that each of 16 samples of *E. verrucosus* collected in B. Goloustnoe settlement in August 2020 contains at least five drug contaminants. Additionally, ibuprofen was detected in samples collected in 2022 (*N* = 44). Thus, the presence of aspirin, acetaminophen, tetracycline, azithromycin and ibuprofen in amphipods was confirmed. However, different levels of contamination and the absence of quantitative data do not allow us to assume pathways for the transformation and migration of active pharmaceutical ingredients through the ecosystem.

The presence of active pharmaceutical ingredients in the endemic amphipods of *E. verrucosus* species can be explained by their entry into the ecosystem through sewage or groundwater [32]. For example, paracetamol (acetaminophen), ibuprofen and azithromycin are of synthetic origin, and are widely used in medicine, veterinary medicine and households [33,34,35,36].

Detection of these molecular ions of drugs in amphipods may indicate their regular entry into domestic wastewater in unchanged form. For instance, acetaminophen has been previously detected in water [30], suggesting that this active ingredient is regularly released into the ecosystem of Lake Baikal. The presence of other pharmaceutical contaminants, such as dimetridazole, metronidazole, and amikacin, may be attributed to the successful use of antibiotics in livestock to treat and prevent infectious diseases [37,38,39,40].

In addition to their synthetic origin, active ingredients of drugs can also be obtained naturally. For example, acetylsalicylic acid is a derivative of salicylic acid, which is the active ingredient in willow [38], raspberry, blackberry and blackcurrant extracts [41]. Thus, aspirin can enter the water not only with sewage but also with particles of bark and leaves of plants containing salicylate. Thus, amphipods, being phyto- and detritophagous [42], could consume these natural products with food, as Baikal algae, whose chemistry of natural products is not studied.

Additionally, some naturally occurring active ingredients can be detected due to symbiotic microorganisms living in the gastrointestinal tract or hemolymph of amphipods. These microorganisms are capable of producing natural compounds. For example, tetracycline is a semi-synthetic antibiotic and can also be produced by actinobacteria related to *Streptomyces aureofaciens* and *S. rimosus*. These microorganisms are regular symbionts of amphipods [43,44,45]. Notably, oxytetracycline, doxycycline and chlortetracycline, identified during the study, also belong to the tetracycline group. Spiramycin refers to natural antibiotics in the macrolide group and is synthesized by actinobacteria of species *Streptomyces ambofaciens* [46,47].

Thus, our study demonstrates that the amphipods of Lake Baikal can uptake pharmaceutical contaminants in their environment. Although the stress response of Baikal-endemic amphipods to heavy metals [48,49], natural organic matter [50], or model organic pollutant [51] is well studied, the relationship of Baikal amphipods to pharmaceutical contaminants has never been investigated. Additionally, pharmaceutical pollutants can have toxic effects on aquatic organisms [52,53,54] and induce oxidative stress [55,56]. Pharmaceutical pollutants can also lead to a decrease in biodiversity, which is especially relevant for the ancient Lake Baikal [57]. The problem acquires particular urgency and significance in view of the tolerance of blue-green algae to ibuprofen, a typical pharmaceutical pollutant, and the effects of blue-green algae growth regularly observed in Lake Baikal [32]. Thus, assessing pharmaceutical contamination of ancient ecosystems and studying the response of endemic organisms can help answer global questions, including those related to climate change, antibiotic resistance, and the overall human impact on the planet.

## 4. Materials and Methods

Amphipods (Amphipoda, Crustacea) were chosen as the subject of our study. Amphipods are one of the most diverse groups of organisms in Lake Baikal, with taxonomic diversity and different ecological preferences. To date, the endemic fauna of Baikal amphipods is represented by more than 350 endemic species and subspecies [42].

For this study, we selected amphipods of the species *Eulimnogammarus verrucosus* (Gerstf., 1858). Samples were collected with a hydrobiological net on the stone littoral at a depth of 0.1–0.5 m near Bolshoe Goloustnoe settlement in August 2020 and 2022. Bolshoe Goloustnoe is a settlement located 120 km from Irkutsk, and it is a representative Baikal settlement with moderate anthropogenic, agricultural and touristic load. Each amphipod was fixed at the sampling site into disposable and sterile Eppendorf microtubes. Each amphipod was placed in a separate tube and frozen at −20 °C.

In the laboratory, each individual was weighed using an analytical scale (Sartogosm CE224-C, St. Petersburg, Russia). The wet weight of the amphipods was 0.1–0.7 g and their average weight was 0.28 g. The samples were further homogenized using vibration ball mill in the presence of acetonitrile at the ratio of 1:10 (1—weight of amphipods, g; 10—volume of acetonitrile, mL). Each sample contained one individual crustacean. The obtained homogenate was centrifuged for 10 min at 3000× *g* rpm (Armed LC04B, St. Petersburg, Russia). Then 800 μL of supernatant was transferred to microtubes and protein precipitation was performed by adding 10% trichloroacetic acid solution in a volume of 80 μL. Then, microtubes were centrifuged again for 10 min at 16,000× *g* rpm (Biosan Microspin-12, Riga, Latvia). The prepared samples were stored at +4 °C. Prior to analysis, the samples were filtered through a 13 mm diameter, 45 µm pore size syringe filter with PVDF membrane, and then 100 µL of the filtrate was transferred into chromatographic vials. The extracts were diluted with 900 µL of acetonitrile [58,59,60].

In this study, we prepared standardized samples of active pharmaceutical ingredients on our own by extracting them from drugs. For qualitative analysis, solutions of aspirin, acetaminophen, ibuprofen, tetracycline, and azithromycin were made from crushed tablets. Analytical standards of ibuprofen (11559-2020, NCAS, Moscow, Russia) and azithromycin (11570-2020, NCAS, Moscow, Russia) were used as additional reference positive control. The reference positive control of ibuprofen was used in concentration of 72 ng/mL and added to extracts of amphipods. Methyl alcohol served as the solvent. The solutions prepared from analytical standards and from crushed tablets were used to determine correct ionization and fragmentation parameters for molecules of the analyzed active ingredients [61,62]. Additionally, these solutions were used to assess the suitability of chromatographic systems and to establish retention times. To avoid false-positive results, we used the method involving the analysis of natural sample, analytical control and the analysis of a sample with a standard control added to it. The negative control was a mixture of solvents and rinses that are used to rinse Eppendorf tubes and filters [62,63,64].

A qualitative analysis of the content of other active ingredients was conducted using the multiple reaction monitoring (MRM) mode [65,66,67]. The MRM transitions of the studied drugs were described using GOST standards (Gosudarstvennyy Standart, a set of international technical standards maintained by the Euro-Asian Council for Standardization, Metrology and Certification for the Commonwealth of Independent States), which are employed for the control of active ingredients in composition of food products and food raw materials (Table 3).

Screening for the presence of active ingredients in samples of Baikal-endemic amphipods was performed using the Agilent Infinity II (2019) chromatography mass spectrometry system with an Agilent 6470B triple quadrupole mass spectrometry detector (Supplementary Materials S1, Figure S11). Agilent Poroshell C18 chromatographic column 2.1 × 50 mm; column temperature −30 °C. Table 4 presents the program for HPLC separation used for the general screening (qualitative data). Table 5 presents the program for HPLC separation of samples containing ibuprofen (quantitative data). Mass spectrometer setup program: ion source gas temperature: 300 °C; ion source gas flow: 5 L/min; nebuliser: 45 psi; drying gas temperature: 250 °C; drying gas flow: 11 L/min; capillary voltage: 3500 V; sample volume: 1 µL.

During the study, over 60 samples of individually collected amphipods were analyzed. In total, over the period 2020–2024, more than 200 samples of amphipods were collected and analyzed. Here, we present the first structured data related to the first qualitative data characterizing pharmaceutical contamination detected in amphipods species of *E. verrucosus*, collected in the settlement of BS. Goloustnoe in August 2020 (N = 16) and 2022 (N = 44). Each analyzed sample contained one individual crustacean. The analysis of each sample was conducted three times.

The statistical processing was performed in Past software (V4.03) using the Kruskal–Wallis *H* test (one-way ANOVA on ranks as a non-parametric method) for the analysis of qualitative data. The Bonferroni sequential significance model was used. One-way ANOVA, *t*-test and F-test were used to analyze quantitative data. The effects of factors “year of sampling” and “wet weight of amphipods” were reported based on two-way ANOVA. To determine which group means were significantly different from each other, Tukey’s post-hoc tests were performed (Supplementary Materials S2). Differences between the mean values of the parameters were considered significant at *p* ≤ 0.05.

## 5. Conclusions

Our study reveals that endemic amphipods (*E. verrucosus*) in Lake Baikal are contaminated with active pharmaceutical ingredients and this is the first report showing that invertebrates in Lake Baikal contain such contaminants. The presence of ibuprofen, acetaminophen, tetracycline and other pharmaceuticals may have a negative impact on the endemic organisms of Lake Baikal. For this reason, monitoring the concentrations of these pharmaceuticals and investigating their effects on Lake Baikal organisms are necessary.

## Figures and Tables

**Table 1:** The presence of active of pharmaceutical ingredients in samples of individual amphipods of *E. verrucosus* species collected in August 2020.

	Azithromycin	Tetracycline	Acetaminophen	Ibuprofen	Aspirin
1	−	−	−	+	+
2	−	−	−	−	−
3	+	+	+	+	+
4	+	+	+	+	+
5	−	−	−	−	−
6	−	+	+	−	−
7	−	−	−	−	−
8	+	+	+	−	−
9	+	+	+	+	+
10	+	+	+	+	+
11	+	+	+	+	+
12	+	+	+	+	+
13	+	+	+	+	+
14	+	+	+	+	+
15	+	+	+	+	−
16	+	+	+	+	+
Uncontaminated samples, %					
Total	31	25	25	31	37
Contaminated samples, %					
Total	69	75	75	69	63

“+” Presence of active of pharmaceutical ingredients; “−“ Absence of active of pharmaceutical ingredients.

**Table 2:** The presence of active pharmaceutical ingredients in samples of individual amphipods of *E. verrucosus* species collected in August 2020.

	Amikacin	Chlortetracycline	Doxycycline	Oxytetracycline	Dimetridazole	Metronidazole	Spiramycin
1	+	+	+	+	+	+	−
2	−	+	+	+	+	+	−
3	+	+	+	+	+	+	−
4	+	+	+	+	+	+	−
5	+	+	+	+	+	+	−
6	+	+	+	+	+	+	−
7	+	+	+	+	+	+	−
8	+	+	+	+	+	+	−
9	+	+	+	+	+	+	−
10	+	+	+	+	+	+	−
11	+	+	+	+	+	+	−
12	+	+	+	+	−	+	+
13	+	+	+	+	−	+	+
14	+	+	+	+	−	+	−
15	+	+	+	+	−	+	+
16	+	+	+	+	−	+	+
Uncontaminated samples, %							
Total	6	0	0	0	31	0	75
Contaminated samples, %							
Total	94	100	100	100	69	100	25

“+” Presence of active of pharmaceutical ingredients; “−“ Absence of active of pharmaceutical ingredients.

**Table 3:** MRM transitions of active ingredients of drugs.

	Active Pharmaceutical Ingredient	Precursor Ions (m/z)	Product Ions (m/z)	Reference
1	Acetaminophen	152.0	110.0	[68]
2	Amikacin	586.3	425.2	[69]
3	Amoxicillin	366.1	114.1; 208	[70]
4	Ampicillin	350.1	192,1; 106.1	[70]
5	Apramycin	540.3	217.1	[69]
6	Aspirin	179.0	136.8	[71]
7	Azithromycin	749.6	591.6	[72]
8	Chlortetracycline	479.1	462.1	[73]
9	Clarithromycin	748.5	590.3; 158.0	[74]
10	Demeclocycline	465.1	430.1	[73]
11	Dimetridazole	142.1	81.1; 96.1	[70]
12	Doxycycline	445.1	410.0	[73]
13	Erythromycin	734.5	158.0; 576.3	[74]
14	Florfenicol	356.1	185.1; 119.1	[70]
15	Gentamicin	478.3	157.1	[69]
16	Hygromycin B	528.3	352.1	[69]
17	Ibuprofen	205.1	161.1	[75]
18	Lincomycin	407.2	359.2; 126.0	[74]
19	Metronidazole	172.1	128.1; 82.1	[70]
20	Oxytetracycline	461.1	444.2	[73]
21	Spiramycin	422.2	100.9; 174.1	[74]
22	Sulfamethazine	279.1	186.1; 124.1	[70]
23	Sulfapyridine	250.1	184.1; 156.1	[70]
24	Tetracycline	445.0	410.0	[73]
25	Thiamphenicol	353.9	290.0; 184.9	[70]
26	Tylosin	916.5	772.1;174.0	[74]

**Table 4:** HPLC separation program used for general screening.

Time, min	Solution A 100% Milli-Q Water, %	Solution B 100% Acetonitrile, %	Flow Rate, mL/min
0.5	95	5	0.4
1.0	85	15	0.4
6.0	20	80	0.4
8.0	20	80	0.4
9.0	95	5	0.4

**Table 5:** HPLC separation program used to estimate ibuprofen concentration.

Time, min	Solution A 0.1% Acetic Acid Solution, %	Solution B 100% Acetonitrile, %	Flow Rate, mL/min
0.5	60	40	0.4
4.0	2	98	0.4
6.0	2	98	0.4
7.0	60	40	0.4
7.5	60	40	0.4

## Data Availability

The original contributions presented in the study are included in the article (and Supplementary Material), further inquiries can be directed to the corresponding authors.

## Supplementary Materials

The following supporting information can be downloaded at: https://www.mdpi.com/article/10.3390/antibiotics13080738/s1.
