# Supplementary material for: Detection of Pharmaceutical Contamination in Amphipods of Lake Baikal by the HPLC-MS Method
Source: Antibiotics (Basel). 2024 Aug 6;13(8):738. doi: 10.3390/antibiotics13080738 (PMC11350909; doi:10.3390/antibiotics13080738)
Supplement: Supplementary file 1 [file antibiotics-13-00738-s001.zip › Supplementary Materials S1.pdf]

Supplementary Materials

Detection of Pharmaceutical Contamination in Amphipods of Lake Baikal by the HPLC-MS Method

Tamara Y. Telnova †, Maria M. Morgunova †, Sophie S. Shashkina, Anfisa A. Vlasova, Maria E. Dmitrieva, Victoria N. Shelkovnikova, Ekaterina V. Malygina, Natalia A. Imidoeva, Alexander Y. Belyshenko, Alexander S. Konovalov, Evgenia A. Misharina and Denis V. Axenov-Gribanov *

Research Department, Biological and Soil Faculty, Irkutsk State University, Irkutsk 664003, Russia

* Correspondence: denis.axengri@gmail.com; Tel.: +7-950-065-84-55

† These authors contributed equally to this work.

A

B

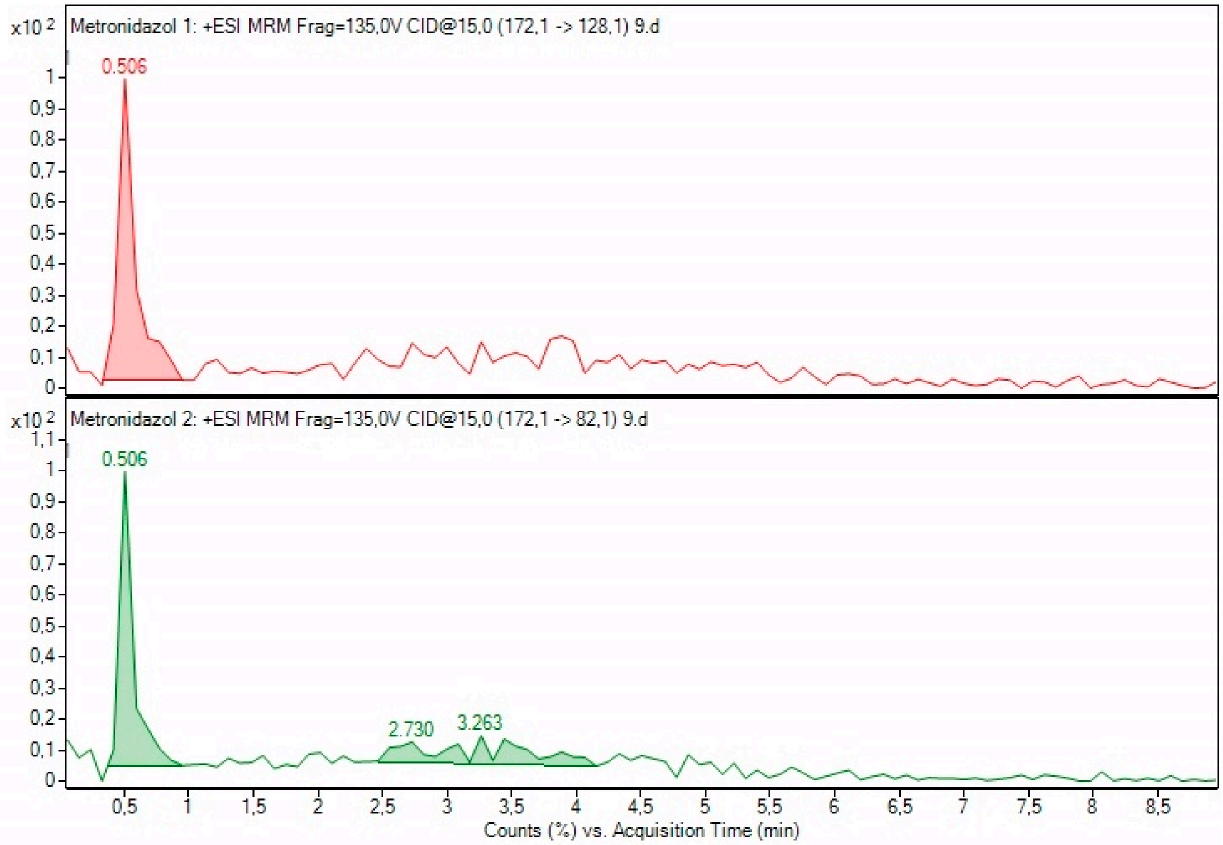

Figure S1. Typical raw data chromatogram of metronidazole detected in samples of amphipod *E. verrucosus*. MRM transitions: A – 172.1 → 128.1; B – 172.1 → 82.1

A

B

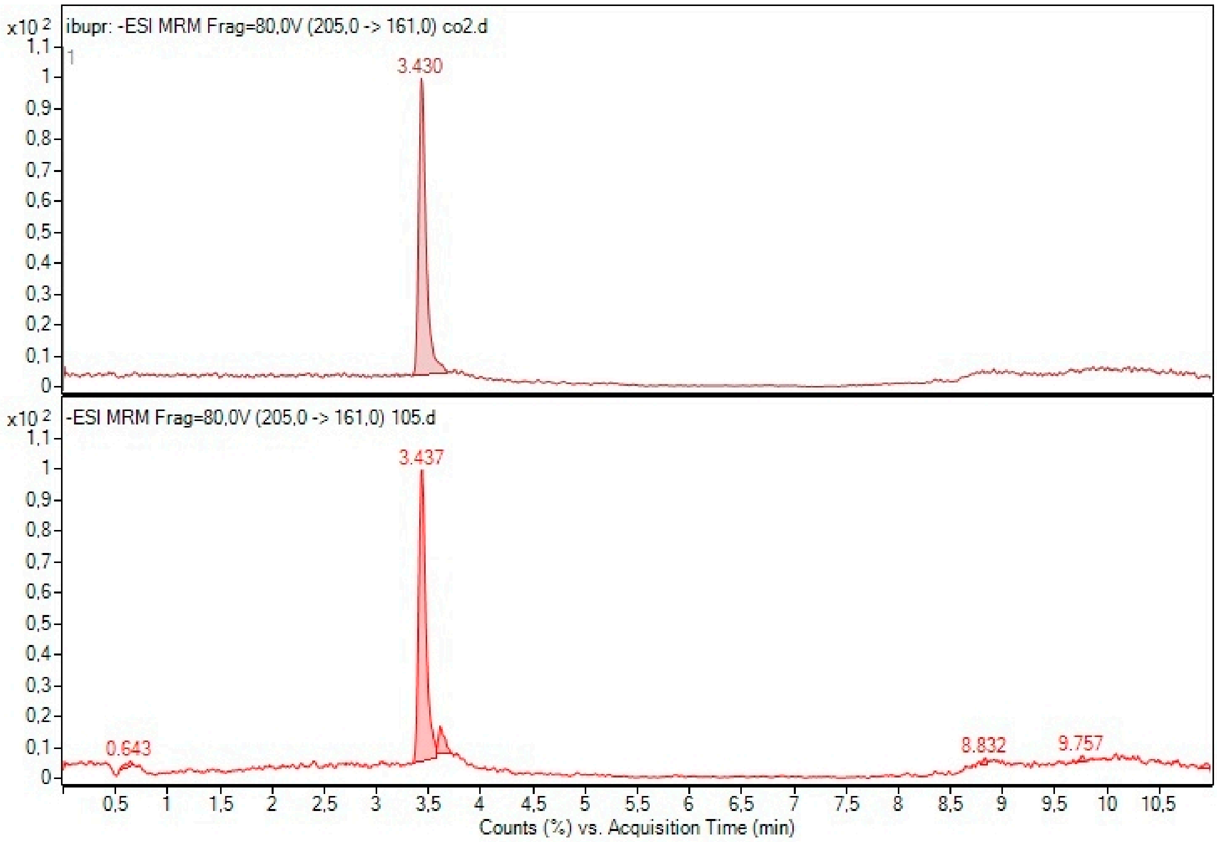

Figure S2. Typical raw data chromatogram of ibuprofen detected in samples of amphipod *E. verrucosus*; A – analytical standart of ibuprofen; B – ibuprofen in sample of amphipod *E. verrucosus*. MRM transitions: 205.0 → 161.0

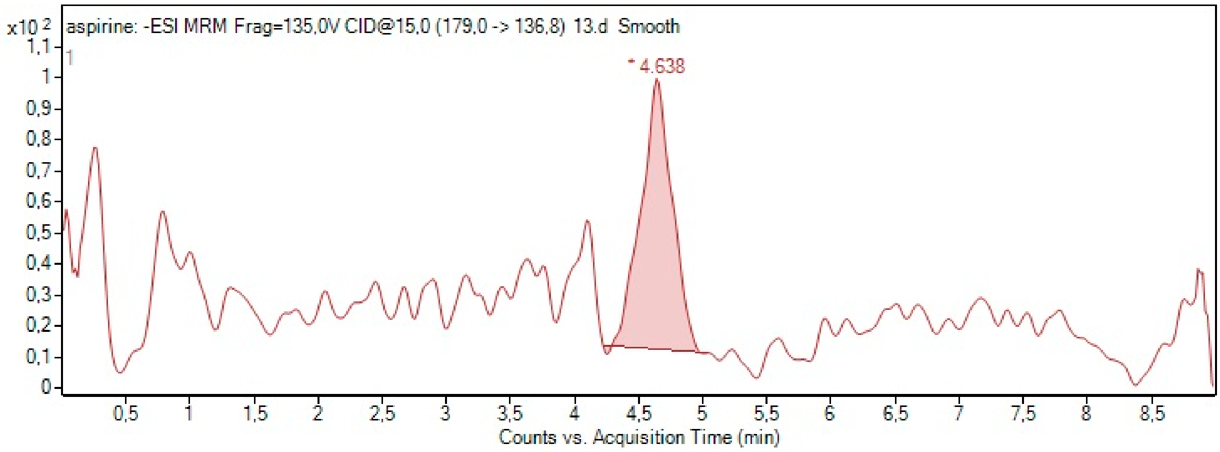

Figure S3 Typical raw data chromatogram of aspirine detected in samples of amphipod *E. verrucosus*. MRM transitions: 179.0 → 136.8

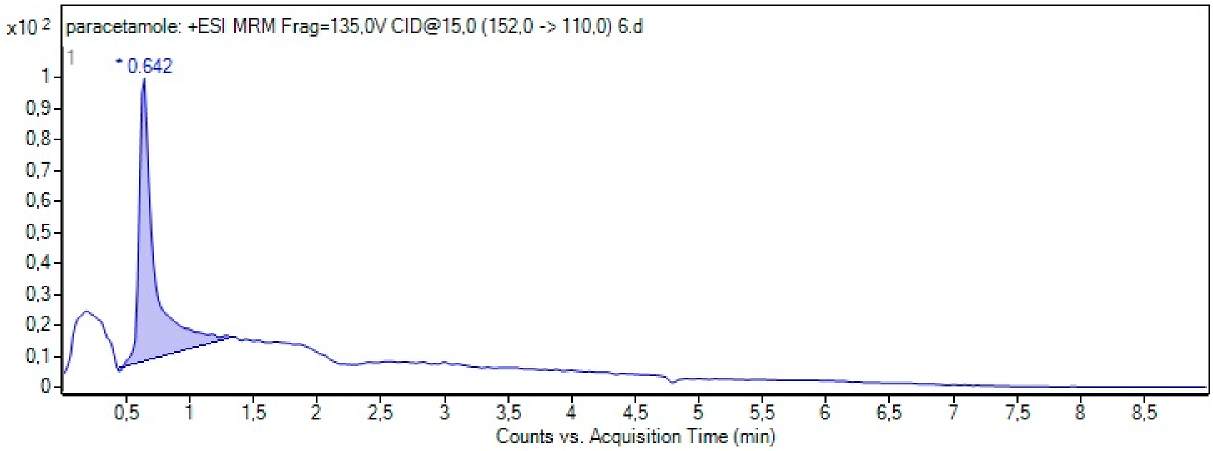

Figure S4. Typical raw data chromatogram of acetaminophen detected in samples of amphipod *E. verrucosus*. MRM transitions: 152.0 → 110.0

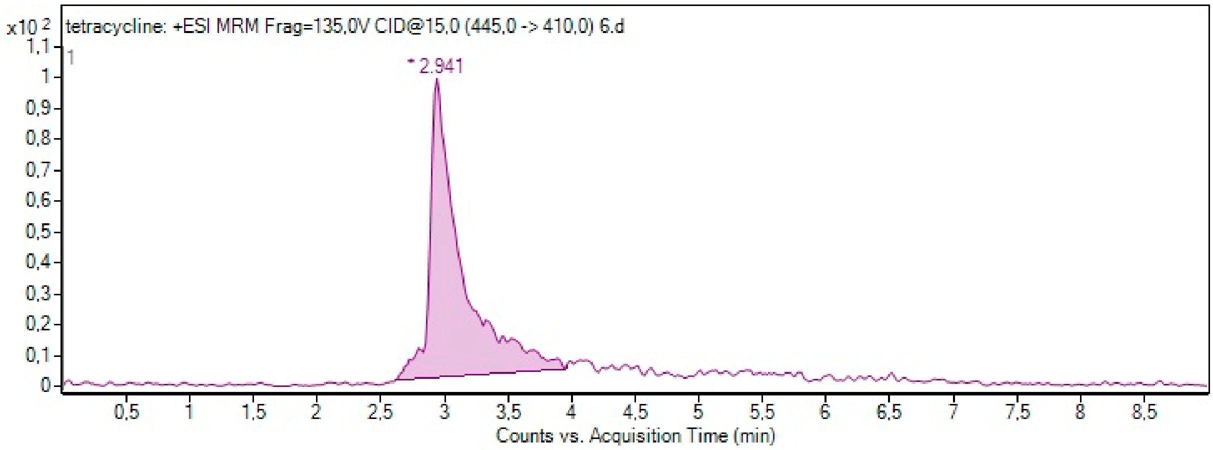

Figure S5. Typical raw data chromatogram of tetracycline detected in samples of amphipod *E. verrucosus*. MRM transitions: 445.0 → 410.0

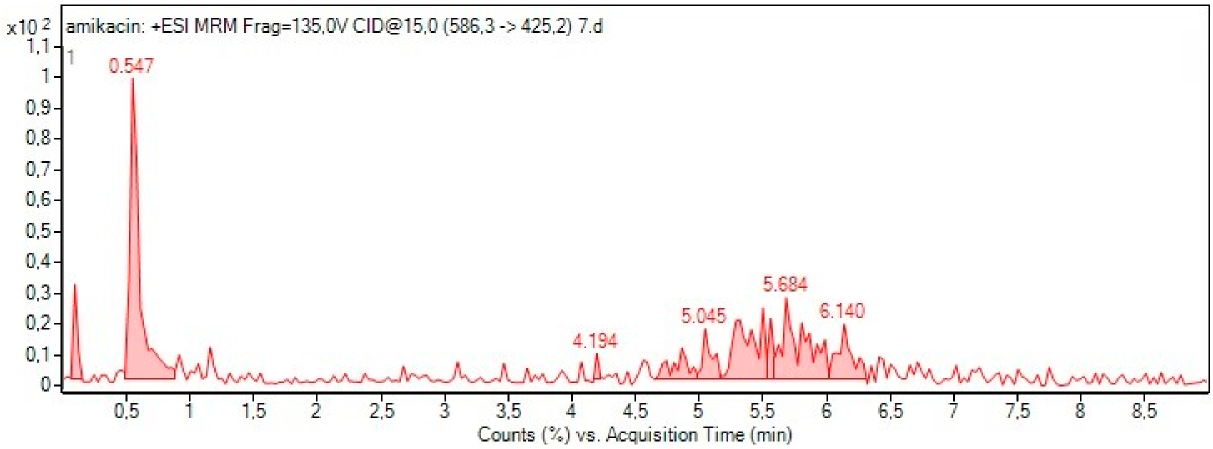

Figure S6. Typical raw data chromatogram of amikacin detected in samples of amphipod *E. verrucosus*. MRM transitions: 586.3 → 425.2

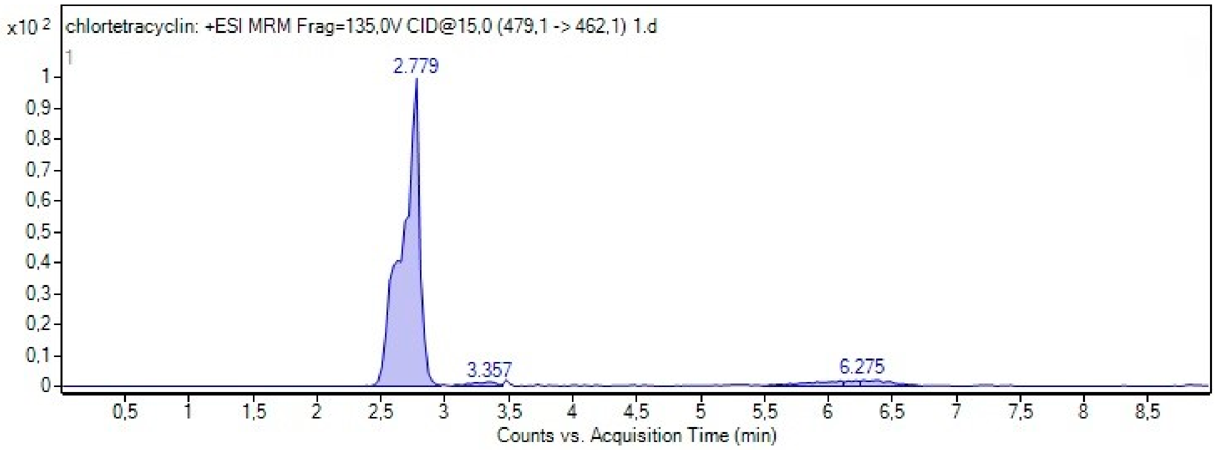

Figure S7. Typical raw data chromatogram of chlortetracycline detected in samples of amphipod *E. verrucosus*. MRM transitions: 479.1 → 462.1

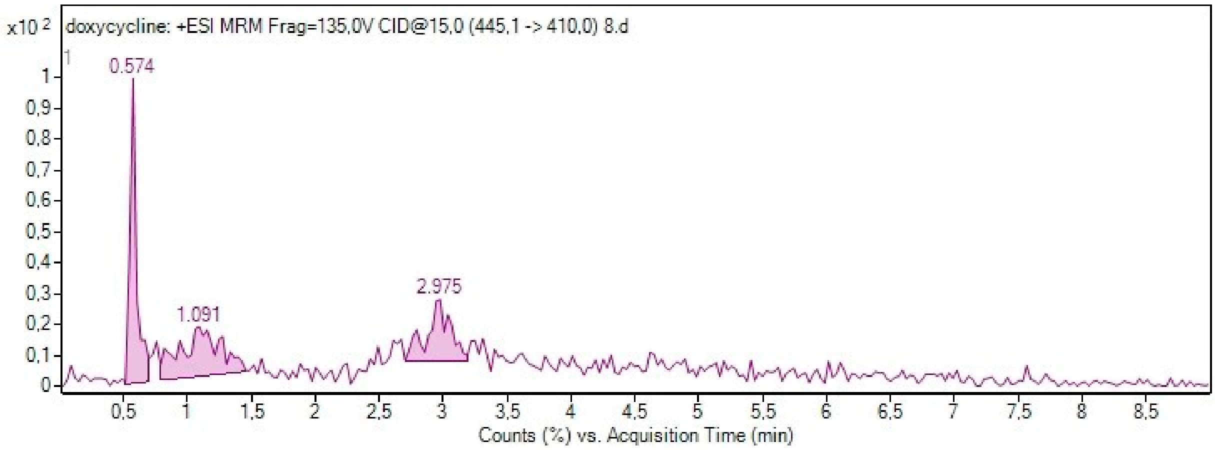

Figure S8. Typical raw data chromatogram of doxycycline detected in samples of amphipod *E. verrucosus*. MRM transitions: 445.1 → 410.0

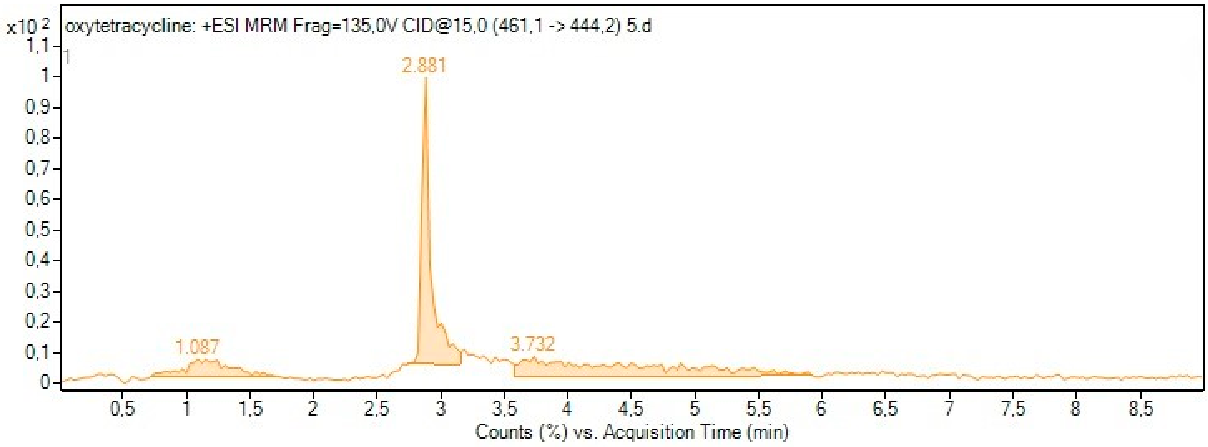

Figure S9. Typical raw data chromatogram of oxytetracycline detected in samples of amphipod *E. verrucosus*. MRM transitions: 461.1 → 444.2

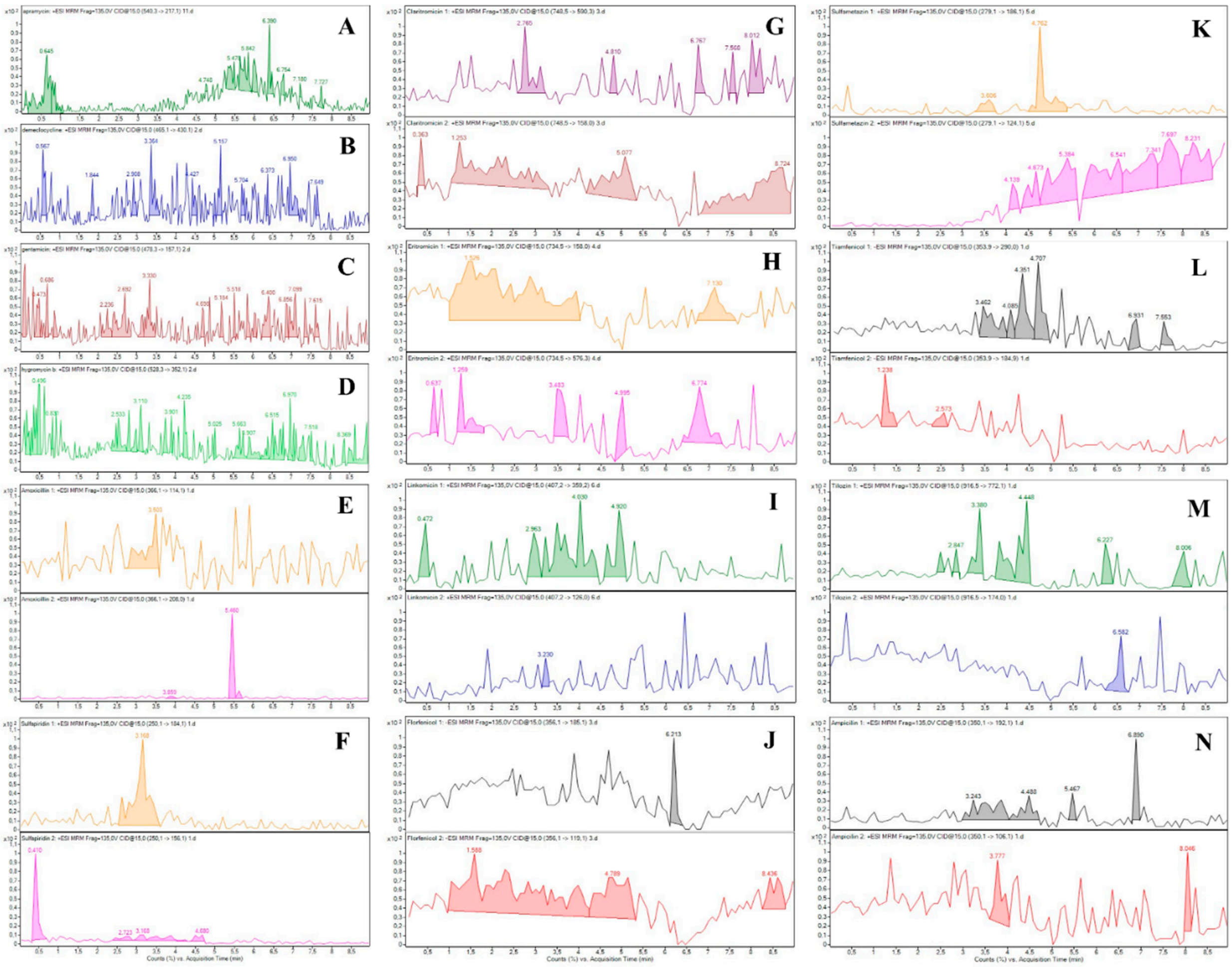

Figure S10. Typical raw data chromatogram of samples where pharmaceutical contaminants were not detected: A – apramycin; B – demeclocycline; C – gentamicin; D – hygromycin B; E – amoxicillin; F – sulfapyridine; G – clarithromycin; H – erythromycin; I – lincomycin; J – florfenicol; K – sulfamethazine; L – thiamphenicol; M – tylosin; N – ampicillin.

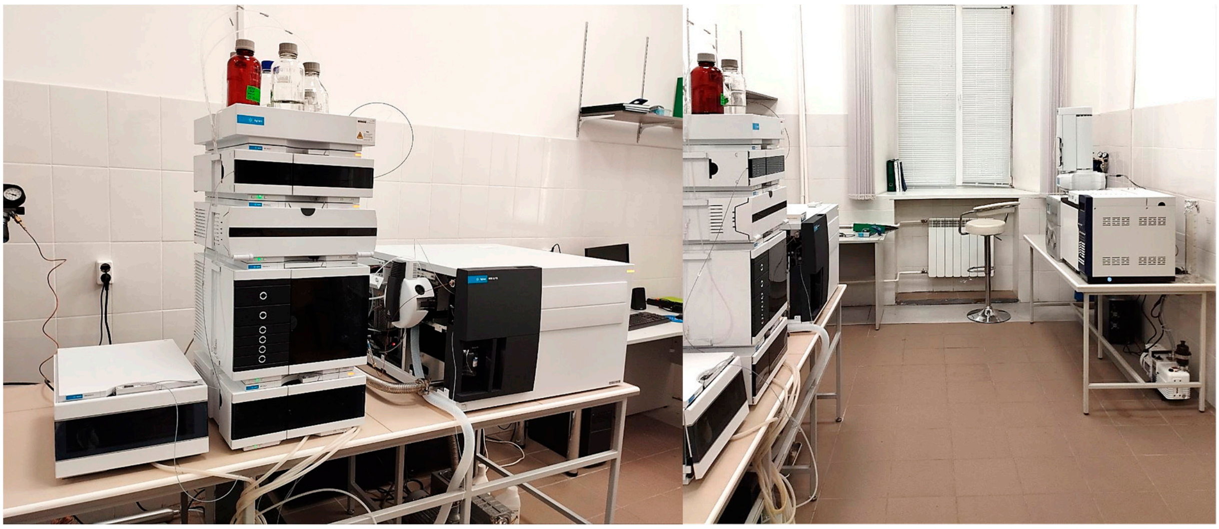

Figure S11. HPLC-MS Agilent and chromatographic laboratory of Irkutsk state university
